# Sensory Evidence Accumulation Using Optic Flow in a Naturalistic Navigation Task

**DOI:** 10.1523/JNEUROSCI.2203-21.2022

**Published:** 2022-07-06

**Authors:** Panos Alefantis, Kaushik Lakshminarasimhan, Eric Avila, Jean-Paul Noel, Xaq Pitkow, Dora E. Angelaki

**Affiliations:** ^1^Center for Neural Science, New York University, New York, New York 10003; ^2^Center for Theoretical Neuroscience, Columbia University, New York, New York 10027; ^3^Department of Neuroscience, Baylor College of Medicine, Houston, Texas 77030; ^4^Department of Electrical and Computer Engineering, Rice University, Houston, Texas 77005-1892; ^5^Center for Neuroscience and Artificial Intelligence, Baylor College of Medicine, Houston, Texas 77030; ^6^Tandon School of Engineering, New York University, New York, New York 11201

**Keywords:** closed loop, naturalistic, navigation, nonhuman primates, sensory accumulation, virtual reality

## Abstract

Sensory evidence accumulation is considered a hallmark of decision-making in noisy environments. Integration of sensory inputs has been traditionally studied using passive stimuli, segregating perception from action. Lessons learned from this approach, however, may not generalize to ethological behaviors like navigation, where there is an active interplay between perception and action. We designed a sensory-based sequential decision task in virtual reality in which humans and monkeys navigated to a memorized location by integrating optic flow generated by their own joystick movements. A major challenge in such closed-loop tasks is that subjects' actions will determine future sensory input, causing ambiguity about whether they rely on sensory input rather than expectations based solely on a learned model of the dynamics. To test whether subjects integrated optic flow over time, we used three independent experimental manipulations, unpredictable optic flow perturbations, which pushed subjects off their trajectory; gain manipulation of the joystick controller, which changed the consequences of actions; and manipulation of the optic flow density, which changed the information borne by sensory evidence. Our results suggest that both macaques (male) and humans (female/male) relied heavily on optic flow, thereby demonstrating a critical role for sensory evidence accumulation during naturalistic action-perception closed-loop tasks.

**SIGNIFICANCE STATEMENT** The temporal integration of evidence is a fundamental component of mammalian intelligence. Yet, it has traditionally been studied using experimental paradigms that fail to capture the closed-loop interaction between actions and sensations inherent in real-world continuous behaviors. These conventional paradigms use binary decision tasks and passive stimuli with statistics that remain stationary over time. Instead, we developed a naturalistic visuomotor visual navigation paradigm that mimics the causal structure of real-world sensorimotor interactions and probed the extent to which participants integrate sensory evidence by adding task manipulations that reveal complementary aspects of the computation.

## Introduction

To survive in a perpetually uncertain, volatile world, we must make sequential decisions within a limited time horizon. To succeed, we accumulate information from our noisy environment to inform decisions for desirable outcomes. Sensory evidence accumulation is considered a hallmark of perceptual decisions and is used to reduce uncertainty in favor of optimal potential action. However, insights about how the brain integrates this come largely from simple laboratory tasks in which sensory cues are discrete and/or have statistics that remain stationary over time (Glass and Pérez, 1973; de Bruyn and Orban, 1988; Snowden and Braddick, 1990; Kim and Shadlen, 1999; Gold and Shadlen, 2000; Watanabe and Kikuchi, 2006; Gu et al., 2008; Liu and Pleskac, 2011; de Lafuente et al., 2015; Drugowitsch et al., 2015; Hou et al., 2019). In reality, statistics of sensory inputs can change continuously depending on actions taken, creating a closed-loop interaction between perception and action. To study computations that underlie these dynamic behaviors, we must employ tasks that resemble moderately complex naturalistic environments, striking a balance between recapitulating the rich dynamics of the world and exerting control over task variables.

One example of real-world sequential action-perception interactions is path integration, an ethological behavior that involves integrating optic flow cues generated by one's self-motion (humans, Ellmore and McNaughton, 2000; Kearns et al., 2002; Butler et al., 2010; Wiener et al., 2016; insects, Collett and Collett, 2017, 2000; Heinze et al., 2018; https://era.ed.ac.uk/handle/1842/28909; rodents, Kautzky and Thurley, 2016; Thurley and Ayaz, 2017; Campbell et al., 2018). This helps one maintain a sense of position, even when explicit position cues are unavailable (Loomis et al., 1999; Collett and Collett, 2000; Etienne and Jeffery, 2004). For example, optic flow indicates a change in angular position during rotational self-motion; during translation, its radial pattern provides information to estimate changes in displacement. Although optic-flow-based path integration is a real-world behavior likely involving time integration of self-motion velocity cues, it is seldom exploited as a sensory evidence accumulation task, where observations are continuously combined with predictions of the internal model of sensorimotor dynamics; an approach that is distinct from the accumulation-to-bound process commonly studied in decision-making.

Instead, primate studies of optic flow evidence accumulation have been limited to passive viewing laboratory tasks, where sensory cues and actions are discrete (e.g., in two alternative forced choices) and intermittent (e.g., end of trial; Gu et al., 2008; Drugowitsch et al., 2015; Hou et al., 2019). This is also true for visual motion generally, a classical stimulus for sensory-based decision-making studies (Gold and Shadlen, 2007). Such laboratory tasks differ strikingly from reality. Do the principles learned there extend into naturalistic behaviors?

To address this, we developed a naturalistic visuomotor virtual navigation task where subjects used a joystick to navigate to a target on the ground plane using optic flow cues. Previous work using this paradigm tested whether self-motion estimates are integrated optimally to compute position (Lakshminarasimhan et al., 2018a; Noel et al., 2020), and whether the resulting beliefs about position may reflect in eye movements (Lakshminarasimhan et al., 2020). However, we do not know the extent to which those estimates originate from integrating optic flow. To exploit this task in the context of sensory-based continuous control, we tested whether subjects integrate their displacement cued by optic flow and rule out other navigation strategies that rely solely on an internal model of control dynamics. We adjudicate between the above alternatives using three task variations. First, we incorporated optic flow perturbations in random directions and amplitudes to test subjects' dynamic compensation. Second, we manipulated the joystick gain, challenging subjects to adjust their actions to navigate to the target. Third, we manipulated the optic flow density, varying the informativeness of sensory cues. We show that both macaques and humans rely on optic flow to perform this task, thus proving cross-species consistency, important for future exploration of the underlying neural substrates.

## Materials and Methods

### Human and animal participants

Three rhesus macaques (all male, 7–8 years old) participated in the experiments. All surgeries and experimental procedures were approved by the Institutional Animal Care and Use Committee and were in accordance with National Institutes of Health guidelines (Baylor College of Medicine, Houston, Texas).

Additionally, four distinct groups of human subjects participated in the three variants of the experiment, nine human subjects (six males, three females, age 20–30 years) in the perturbation variant, seven (four males, three females, age 18–30 years) in the gain variant, six (four males, two females, age 20–30 years) in the density variant, and 11 (six males, five females, age 21–30 years) in the time-varying density variant. All human subjects were unaware of the purpose of the study and signed an approved consent form before their participation in the experiment.

### Experimental setup

At the beginning of each experimental session, monkeys were head fixed and secured in a primate chair placed on top of a platform (Kollmorgen). A three-chip DLP projector (Christie Digital Mirage 2000) was mounted on top of the platform and rear projected images onto a 60 × 60 cm tangent screen ∼30 cm in front of the monkey. The projector was capable of rendering stereoscopic images generated by an OpenGL accelerator board (Nvidia Quadro FX 3000G). Spike2 software (Power1401 MkII data acquisition system, Cambridge Electronic Design) was used to record joystick and all event and behavioral markers for off-line analysis at a sampling rate of 833$\frac{1}{3}$ Hz.

All stimuli were generated and rendered using C++ Open Graphics Library (OpenGL) by continuously repositioning the camera based on joystick inputs to update the visual scene at 60 Hz. The virtual camera was positioned at a height of 10 cm above the ground plane. Spike2 software (Power1401 MkII data acquisition system, Cambridge Electronic Design) was used to record and store the target location (*r*, θ), the position of the subject ($\tilde{r}$, $\tilde{\theta }$).

For humans, all other aspects of the setup were similar to the one used for monkeys, but with subjects seated 67.5 cm in front of a 149 × 127 cm^2^ (width × height) rectangular screen and with the virtual camera placed 100 cm above the ground plane. The time-varying optic flow density manipulation was presented in virtual reality (HTC Vive) and built in the Unity development tool. The participant's head was fixed on the chair using an adjustable CIVCO FirmFit 490 Thermoplastic face mask.

### Behavioral task

**Figure 1.**
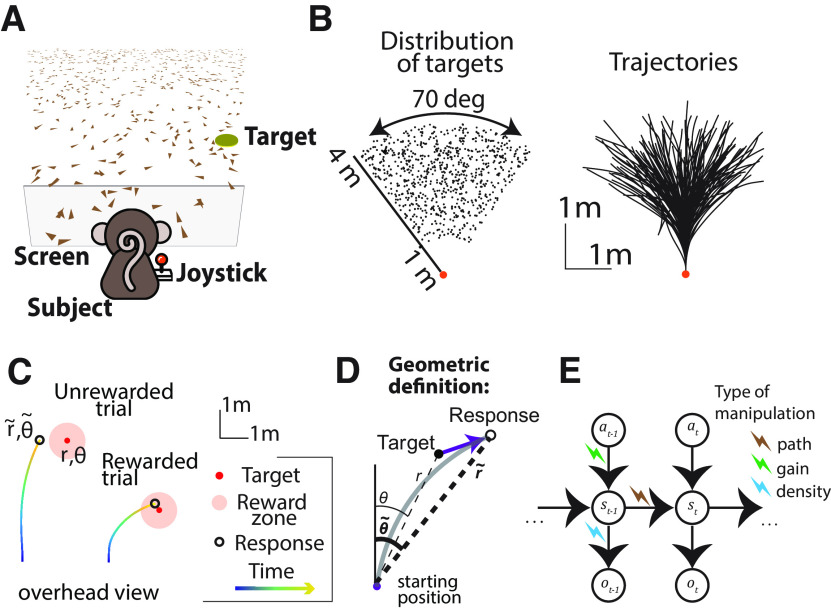
The firefly task. ***A***, Monkeys and humans use a joystick to navigate to a cued target (yellow disk) using optic flow cues generated by ground plane elements (brown triangles). The ground plane elements appeared transiently at random orientations to ensure that they cannot serve as spatial or angular landmarks. ***B***, Left, Overhead view of the spatial distribution of target positions across trials for monkey subjects. Positions were uniformly distributed within subjects' field of view. The actual range of target distances and angles was larger for human subjects (see above, Materials and Methods). Right, Movement trajectories of one monkey during a representative subset of trials. Orange dot denotes starting position. ***C***, Example trials showing incorrect (left) and correct (right) responses. Note that subjects had to stop within a ±0.6 m zone to receive reward. ***D***, Geometric definition of analysis variables. The gray solid line indicates an example trajectory. The target and response distance and angle relative to the starting position of the participant are given by *r*, θ (thin lines) and *r̃*, $\tilde{\theta }$ (thick lines), respectively. ***E***, Schematic representation of the Markov decision process that governs self-motion sensation. a, Action (joystick input); s, state (velocity, position); o, sensory observations. Subscripts denote time indices. We used three manipulations to alter the causal dependency of the variables involved in the navigation process, visual perturbations (brown bolt), gain manipulations (green bolt), density manipulation (blue bolt).

Subjects used an analog joystick (M20U9T-N82, CTI electronics) with two degrees of freedom and a square displacement boundary to control their linear and angular speed in a virtual environment. This virtual world was composed of a ground plane whose textural elements had a limited lifetime (∼250 ms) to avoid serving as landmarks (Fig. 1). The ground plane was circular with a large radius of 70 m (near and far clipping planes at 5 cm and 4000 cm, respectively), and the subject was positioned at its center at the beginning of each trial. Each texture element was an isosceles triangle (base times height, 8.5 × 18.5 cm^2^) which was randomly repositioned and reoriented anywhere in the arena at the end of its lifetime, making it impossible to use as a landmark. The maximum linear and angular speeds were initially fixed to υ_max_ = 2 m/s and ω_max_ = 90°/s, respectively, and then varied by a factor of 1.5 and/or 2.0. The density of the ground plane was either held fixed at ρ = 2.5 elements/m^2^ or varied randomly between two values (ρ = 2.5 elements/m^2^ and ρ = 0.1 elements/m^2^) in a subset of recording sessions (see below). The stimulus was rendered as a red-green anaglyph and projected onto the screen in front of the subject's eyes. Except when wearing a virtual reality headset, subjects wore goggles fitted with Kodak Wratten filters (red no. 29 and green no. 61) to view the stimulus. The binocular cross-talk for the green and red channels was 1.7 and 2.3%, respectively. Target positions were uniformly distributed within the subjects' field of view with radial distances and angles that varied from 1 to 4 m and –35 to 35°, respectively, for monkey experiments. In the human experiments, the radial distance and the angle of the targets varied from 1 to 6 m and –40 to 40°, respectively. In the time-varying optic flow experiment in humans the targets varied from –30 to 30° in eccentricity but were always presented at 3 m in radial distance.

Monkeys received binary feedback at the end of each trial. They received a drop of juice if, after stopping, they were within 0.6 m away from the center of the target; otherwise, no juice was provided. The fixed reward boundary of 0.6 m was determined using a staircase procedure before the experiment to ensure that monkeys received reward in approximately two-thirds of the trials. Human subjects did not receive feedback during the experiment, with the exception of three subjects, for which feedback consisted of a bull's-eye pattern consisting of six concentric circles (with the radius of the outermost circle being continuously scaled up or down by 5%, according to the one-up, two-down staircase procedure), displayed with an arrowhead indicating the target location on the virtual ground. The arrowhead presented was displayed either in red or green to denote whether the participant's response had occurred within the outermost rewarded circle (Lakshminarasimhan et al., 2020).

### Movement dynamics

Let $s_{t}$, $o_{t}$, and $a_{t}$ denote the participant's state (velocity), observation (optic flow), and action (joystick position), respectively. The equations governing movement dynamics in this experiment are as follows:

(1.1) $$s_{t}=Ks_{t-1}+Ga_{t}+\eta_{t}$$

(1.2) $$o_{t}=s_{t}+\varepsilon_{t},$$

where K denotes resistance to change in state, G denotes the gain factor of the joystick controller, $\eta_{t}$ denotes process noise, and $\varepsilon_{t}$ denotes observation noise. In all our experiments, we set K=0 so there was no inertia. Participants can navigate by integrating their velocity to update position x as $x_{t+1}=x_{t}+s_{t}\Delta t$, where Δt denotes the temporal resolution of updates. Note that the experiment involved two degrees of freedom (linear and angular), so the above equation applies to both.

### Behavioral manipulations

**Figure 2.**
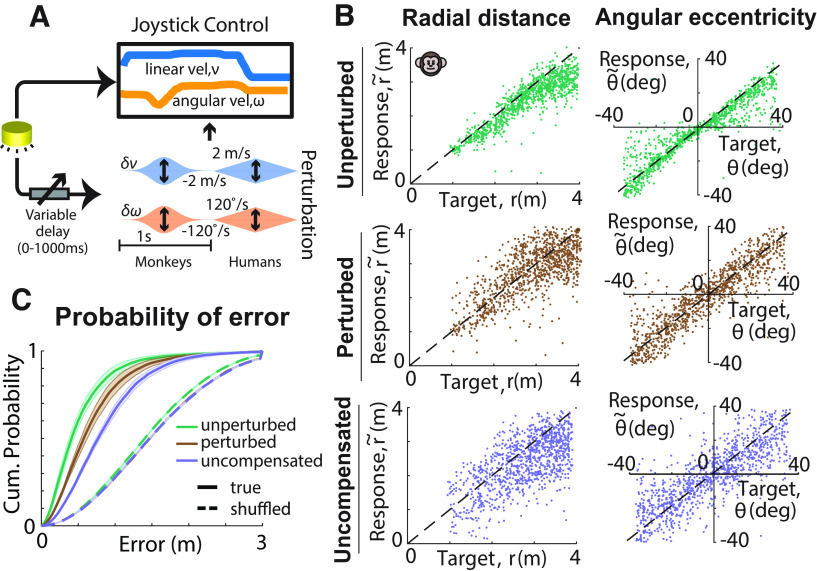
Unpredicted perturbations of optic flow challenge subjects to compensate for them. ***A***, Schematic illustration of the perturbation manipulation; after a variable delay (0–1 s) from movement onset, a fixed duration (1 s) perturbation was added to the subjects' instantaneous self-motion velocity. Perturbations consisted of linear and angular velocity components with Gaussian (monkeys) or triangular (humans) profiles, whose amplitudes varied randomly across trials from −2 to 2 m/s and from −120 to 120°/s for the linear and angular velocities, respectively. ***B***, Representative monkey responses. Left, Comparison of the radial distance $\tilde{r}$ of an example subject's response (final position) against the radial distance r of the target for unperturbed (top, green), perturbed (middle, brown), and the simulated uncompensated case (bottom, purple) trials. Right, Similar comparison for the angular eccentricity of the same subject's response $\tilde{\theta }$ versus target angle θ. Black dashed lines show unity slope. ***C***, Cumulative probability of error magnitude for unperturbed (green), perturbed (brown), and simulated (uncompensated case, purple) trials, averaged across all monkeys. Solid lines and dashed lines represent the values obtained from the actual and shuffled data, respectively. Shaded regions represent ±1 SEM.

In the perturbation (Fig. 2*A*) variant of the experiment, normal trials were interleaved with trials that incorporated transient optic flow perturbations to dislocate subjects from their intended trajectory. Mathematically, such perturbations can be understood as setting the process noise ($\eta_{t}$ in Eq. 1.1) to a nonzero value. For monkeys, the perturbations had a fixed duration of 1 s, a velocity with a Gaussian profile of σ = 0.2, and an amplitude drawn from a uniform distribution from –2 to 2 m/s, and from –120 to 120°/s for the linear and angular velocities, respectively. For monkeys, the perturbations had a fixed duration of 1 s, a Gaussian velocity profile with σ = 0.2, and an amplitude drawn from a uniform distribution between –2 and 2 m/s and –120 and 120°/s for the linear and angular velocities, respectively. For humans, the perturbations had also a fixed duration of 1 s, whereas their velocity profile was an isosceles triangle with height that varied with a uniform distribution from –2 to 2 m/s, and –120 to 120°/s for the linear and angular velocities, respectively. For both humans and monkeys, the perturbation onset time was randomly varied from 0 to 1 s after movement onset.

In the gain manipulation variant (see Fig. 5*A*), we switched the gain factor of the joystick controller (G in Equation 1.1) among 1, 1.5, or 2 for monkeys, and between 1 and 2 for humans. For monkeys, we manipulated joystick control in separate blocks of 500 trials, and the ordering of the blocks was randomized between days. For humans, the gain factor varied randomly between trials. Within each trial, both linear and angular velocities were scaled by the same gain factor.

In the density manipulation variant (see Fig. 6*A*) for monkeys, the density of ground plane elements varied between two values, high (2.5 elements/m^2^) and low (0.1 elements/m^2^). For humans, normal trials were interleaved with trials where the elements constituting the ground plane were completely removed after the target disappeared. Density manipulation effectively changes the information borne by optic flow cues.

### Data analyses

Customized MATLAB code was written to analyze data and to fit models. Depending on the quantity estimated, we report statistical dispersions either using a 95% confidence interval, SD, or SEM. The specific dispersion measure is identified in the portion of the text accompanying the estimates. For error bars in figures, we provide this information in the caption of the corresponding figure.

Across all animals and humans, we regressed (without an intercept term) the response positions of each subject ($\tilde{r}$,$\tilde{\theta }$) against target positions (*r*, θ) separately for the radial (*r* vs $\tilde{r}$) and angular (θ vs $\tilde{\theta }$) coordinates, and the radial and angular multiplicative biases were quantified as the slope of the respective regressions. The 95% confidence intervals were computed by bootstrapping.

### Simulation of uncompensated case (perturbations)

To simulate the uncompensated case responses for each trial with a perturbation, we picked an unperturbed trial with the most similar target position to the perturbed trial. Then, we added the angular and linear perturbation velocities (preserving their timing) to the steering angular and linear velocities of the monkey to simulate an uncompensated stopping position if there was no compensation. Specifically, for each time step t within the window of the perturbation duration, we added the instantaneous linear and angular components of the perturbation $\alpha_{t}$ and $\beta_{t}$ from the perturbed trial to the linear and angular steering velocity $v_{t}$ and $\omega_{t}$ of the chosen target-matched unperturbed trial, respectively. As a result, the total linear (${\tilde{v}}_{t}$) and angular (${\tilde{\omega }}_{t}$) instantaneous velocities of the trial during the simulated perturbation were the following:

(2) $${\tilde{v}}_{t}=v_{t}+\alpha_{t}\mathrm{and}{\tilde{\omega }}_{t}=\omega_{t}+\beta_{t}.$$

The velocity time series of the whole trial were then integrated to produce an uncompensated response with no compensation.

### Receiver operating characteristic analysis

To quantify and compare subject performance across conditions (unperturbed, perturbations, uncompensated case), we performed receiver operating characteristic (ROC) analysis as follows. For each subject, we first calculated the proportion of correct trials as a function of a (hypothetical) reward boundary. In keeping with the range of target distances used, we gradually increased the reward boundary until reaching a value that would include all responses. Whereas an infinitesimally small boundary will result in all trials being classified as incorrect, a large enough reward boundary will yield near-perfect accuracy. To define a chance-level performance, we repeated the above procedure, this time by shuffling the target locations across trials, thereby destroying the relationship between target and response locations. Finally, we obtained the ROC curve by plotting the proportion of correct trials in the original dataset (true positives) against the shuffled dataset (false positives) for each value of hypothetical reward boundary. The area under this ROC curve was used to obtain an accuracy measure for all the subjects.

### Perturbation compensation index (perturbations)

To quantify the compensatory responses of the subjects to the perturbations, we computed the perturbation compensation index (PCI) based on the results of the ROC analysis as follows:

(3) $$\text{PCI}=\frac{{\text{AUC}}_{\text{p}}-{\text{AUC}}_{\text{uc}}}{{\text{AUC}}_{\text{np}}-{\text{AUC}}_{\text{uc}}},$$

where AUC_p_ represents the area under the curve (AUC) for trials with perturbations, AUC_uc_ is the AUC for the group of the uncompensated case responses, and AUC_np_ is the AUC for the unperturbed trials. A value of zero indicates an accuracy equal to the uncompensated case response and thus represents a complete lack of compensation, whereas a value of one indicates an accuracy equivalent to the unperturbed trial and thus represents perfect compensation.

### Dynamic response to perturbations

Although the PCI gives the percentage of compensation with respect to the subjects' stopping locations, it does not capture the dynamic evolution of this response over time. For this reason, we computed the dynamic response to perturbations. For each subject, we estimated a perturbation-specific response by computing the trial-averaged deviation in the subject's self-motion velocity (relative to target-matched, unperturbed trials) at various time lags between 0 and 2 s from perturbation onset, in steps of 6 ms. The rationale behind computing the deviation from target-matched unperturbed trials rather than raw velocities is that we essentially subtract the component of the subject's response that is influenced by target location, yielding only the perturbation-specific component. This deviation is normalized by the perturbation amplitude before trial averaging so that the sign denotes the direction of the response relative to the direction of perturbation, and the amplitude denotes the strength of compensation.

### Gain compensation index (gain manipulation)

To quantify the subjects' responses for different joystick gain conditions, we computed the gain compensation index (GCI), which measures the extent to which the subjects compensated for the changes in gain factor (*g* = [1.5, 2]) with respect to the baseline gain (*g_0_* = 1) as follows:

(4) $$\text{GCI}=\frac{g-b/b_{0}}{g-g_{0}},$$

where *g* and $g_{0}$ denote the modified gain factor and the baseline gain factor, respectively, and *b* and $b_{0}$ correspond to the multiplicative behavioral bias (radial or angular) of the block of trials with the modified gain factor and baseline, respectively. The ratio $b/b_{0}$ captures the change in multiplicative bias for the block of trials where the joystick gain was modified. The terms g and $g_{0}$ were experimental parameters, whereas b and $b_{0}$ were derived from behavior. A ratio equal to one denotes a perfect compensation, whereas a value of zero denotes a complete lack of compensation.

For the GCI was continuously computed for an increasing set of trials (see Fig. 5*E*). This set started with the first 20 trials of the block and increased by 20 trial increments until the end of block (500 trials). Increments of different lengths produced qualitatively similar results.

### Multiple linear regression model (gain manipulation)

To test whether subjects perform spatial (as opposed to temporal) integration, we expressed the basic kinematic equation of velocity as follows: υ=x/t in the form log(t)=log(x)−log (υ), which allowed for the implementation of a multiple linear regression. Following earlier work (Kwon and Knill, 2013), we assume that noise variance is constant in logarithmic scale. To measure the influence of distance and velocity we used the following model:

(5) $$\log\left(T_{i}\right)={\text{w}}_{r}\cdot \log\left(r_{i}\right)+w_{\upsilon }\cdot \text{log}(\upsilon_{i}),$$

where *T*, *r*, and υ are travel duration, target distance, and mean velocity of trial i, respectively.

### Data availability

MATLAB code implementing all quantitative analyses in this study is available at https://github.com/panosalef/fireflyTask. Datasets generated by this study are available at https://gin.g-node.org/panosalef/sensory_evidence_accumulation_optic_flow.

## Results

Macaque and human subjects performed a visual navigation (firefly) task in which they used a joystick to steer to a briefly cued target location in a virtual environment devoid of landmarks (Fig. 1*A*; see above, Materials and Methods). In each trial, a circular target appeared briefly on the ground plane at a random location within the subject's field of view (Fig. 1*B*). Subjects had to navigate to the remembered target location using a joystick to control their linear and angular velocity. The task goal was to stop within the circular reward zone of the target. Unless stated otherwise, feedback was provided immediately after the end of each trial (Fig, 1*C*; see above, Materials and Methods). The virtual ground plane elements were transient and could therefore not be used as landmarks, only to provide optic flow information.

This active control paradigm can be understood as a partially observable Markov decision process (Åström, 1965; Sutton and Barto, 1998; Kwon et al., 2020) in which the sensory observation $o_{t}$ (optic flow) is determined by the current state $s_{t}$ (velocity, position), which depends only on the previous state $s_{t-1}$ and the current action $a_{t}$ (joystick movement) through the control dynamics (Fig. 1*D*; see above, Materials and Methods; Eq. 1.1 and 1.2). To perform this task optimally, subjects must combine their knowledge of the control dynamics with the sensory observation to estimate their current velocity and integrate that estimate over time so they can stop on reaching the reward zone. In principle, however, subjects could choose to ignore sensory inputs and still perform reasonably well by dead reckoning with an accurate internal model of the control dynamics.

In separate sessions, we used three different manipulations of this firefly task to test whether subjects used sensory evidence accumulation, that is, integrated optic flow. The following causal effects of these manipulations on the decision process are illustrated in Figure 1*E*: (1) random perturbations, which imposed an external passive displacement that moved the subjects away from their expected path, disrupting the transition to the desired state (brown bolt); (2) altered gain of the joystick controller from that used during training changed the effect actions induced on the current state (green bolt); and (3) different densities of the ground plane elements manipulated the informativeness of the observations provided by each state (blue bolt). Next, we explore steering responses for each of these experimental manipulations.

### Subjects compensate for unpredictable perturbations

In a random half of the trials, subjects were gradually displaced from their controlled trajectory (visual trajectory perturbation) while steering, challenging them to counter the displacement to reach their goal location. The perturbation began after a random delay (0–1 s) following the subject's movement onset and consisted of independent linear and angular components whose velocity profiles followed a Gaussian (monkeys) or triangular (humans) waveform (see above, Materials and Methods) lasting 1 s. The onset delay and the amplitude of each perturbation were drawn randomly from uniform distributions (Fig. 2A). To reach the target, subjects needed to update their position estimates based on the amplitude and direction of the imposed perturbation, which could only be estimated by sensory integration of visual motion cues (optic flow).

We compared the subjects' responses (i.e., stopping location) in each trial to the corresponding target location separately for unperturbed and perturbed trials. We also simulated responses for an uncompensated case, where subjects steer toward the original target, completely ignoring imposed perturbations. We generated the uncompensated responses by adding the linear and angular velocities of each perturbation to the self-motion velocity profiles of the monkeys in target-matched trials without perturbations (see above, Materials and Methods; Eq. 2). For each condition (unperturbed, perturbed, uncompensated), we calculated the radial distance $\tilde{r}$ and angular eccentricity $\tilde{\theta }$ of the subjects' final position, which were then compared with the initial target distance *r* and angle θ (Fig. 2*B*). Monkeys behaved well on this task, steering appropriately toward the targets in perturbed and unperturbed conditions. As shown with an example monkey session in Figure 2*B*, both radial distance and angular eccentricity of the responses of the monkeys were highly sensitive to target location for both unperturbed and perturbed trials (Table 1). Furthermore, although perturbations decreased the correlation compared with unperturbed trials for both radial distance and angular eccentricity, this decline was less than what would be expected from the uncompensated case. Results in humans were qualitatively similar (Table 1).

**Table 1.** Pearson's correlation coefficients ± SEM between responses of monkeys and humans and target locations (separately for radial and angular components)

		Unperturbed	*p*	Perturbed	*p*	Uncompensated
Monkeys	Radial	0.91 ± 0.03	<10^−6^	0.85 ± 0.04	<10^−6^	0.76 ± 0.05
	Angular	0.73 ± 0.1	<10^−6^	0.59 ± 0.08	4 · 10^−3^	0.33 ± 0.08
Humans	Radial	0.94 ± 0.03	<10^−3^	0.89 ± 0.04	<10^−6^	0.62 ± 0.05
	Angular	0.38 ± 0.05	<10^−6^	0.33 ± 0.1	0.9	0.33 ± 0.08

Correlation coefficients were computed separately across the set of unperturbed (left), perturbed (middle), and hypothetical uncompensated (right) trials. The *p* values between columns indicate the significance of *t* tests for the difference between the correlation coefficients shown in the surrounding columns.

To more directly test whether subjects compensated for the perturbations, we computed the absolute error, the distance between the stopping position and the target, on each trial. In monkeys, errors in trials with perturbations (0.71 ± 0.08 m SD) were larger (*p* < 10^−6^) than those without perturbations (0.55 ± 0.07 m) but smaller than the uncompensated case (0.87 ± 0.08 m, *p* < 10^−6^). In humans, steering accuracy in perturbation trials was not significantly different from that in nonperturbation trials and was significantly better than the uncompensated estimate (3.19 ± 1.61 m SD vs 3.13 ± 1.86 m without perturbations, *p* = 0.61; uncompensated case, 4.21 ± 1.78 m, *p* < 10^−6^). Thus, perturbations decreased steering accuracy relative to unperturbed trials in monkeys (but not humans), but this increase was much less than expected from the uncompensated case (Fig. 2*C*).

**Figure 3.**
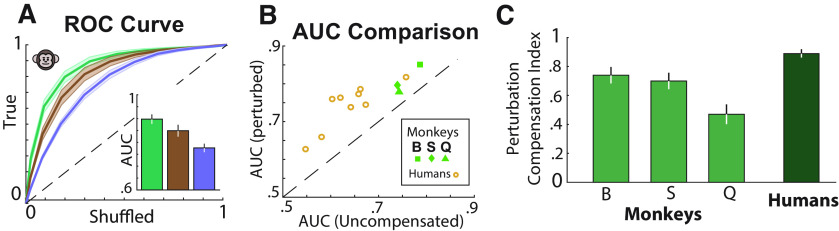
Subjects compensate for unpredicted perturbations of optic flow. ***A***, ROC curves for unperturbed (green), perturbed (brown), and simulated (uncompensated case, purple) trials, averaged across all monkeys. Inset, AUC for the corresponding conditions in ***A***. Shaded regions and error bars denote ±1 SEM. ***B***, Comparison of the AUC of the perturbation (true) and simulated uncompensated trials separately for each monkey and human. ***C***, Bar plot of the mean perturbation compensation index for individual monkeys (light green) and average human subjects (dark green). Error bars denote ±1 SEM.

Because of the slightly larger target distances for humans (see above, Materials and Methods), we couldn't use the mean error magnitude of the subjects to compute accuracy as this ignores differences in task difficulty. Consequently, to quantify performance accuracy across humans and monkeys on a common scale, we adopted the approach of receiver operating characteristic (ROC) to continuous responses. Specifically, for each subject, we computed the actual reward rate and the chance-level reward rate, obtained by shuffling target locations across trials as a function of a hypothetical reward window size (Lakshminarasimhan et al., 2020). We obtained the ROC curves by plotting the actual responses against the responses at chance level and computed the area under the ROC curve (Fig. 3*A*). Chance performance would be reflected by an AUC of 0.5, whereas a perfectly accurate performance would yield an AUC of 1. We compared the area under the curve across conditions for monkeys (mean ± SD, unperturbed, 0.87 ± 0.03; perturbed, 0.83 ± 0.08; uncompensated case, 0.76 ± 0.03) and humans (unperturbed, 0.75 ± 0.03; perturbed, 0.72 ± 0.08; uncompensated case, 0.61 ± 0.08). Although the perturbations reduced response accuracy relative to the unperturbed trials (*t* test, *p* = 0.003), this reduction was much less than expected for uncompensated perturbations (*p* < 10^−6^; Fig. 3*B*). These results show that subjects were able to compensate for optic flow perturbations, supporting the hypothesis that they integrate optic flow for path integration.

To further quantify the extent to which the subjects compensated for perturbations, we computed a PCI (see above, Materials and Methods). A value of zero denotes an accuracy equal to the uncompensated case response and thus a complete lack of compensation, whereas a value of one denotes an accuracy equivalent to the unperturbed trials and thus represents perfect compensation. An examination of the PCI across both monkeys (0.61 ± 0.12; *t* test, *p* < 10^−6^) and humans (0.89 ± 0.1; *t* test, *p* < 10^−6^) showed that, generally, subjects compensate significantly for the perturbations by appropriately adjusting their responses to reach the goal locations, with the humans' PCI values being closer to ideal (Fig. 3*C*). Unlike monkeys, only three of the nine human subjects received end-of-trial feedback during this task (see above, Materials and Methods). Nevertheless, compensation captured by PCI was comparable across both groups (with feedback, 0.86 ± 0.06; without feedback, 0.91 ± 0.12; *t* test, *p* = 0.52).

**Figure 4.**
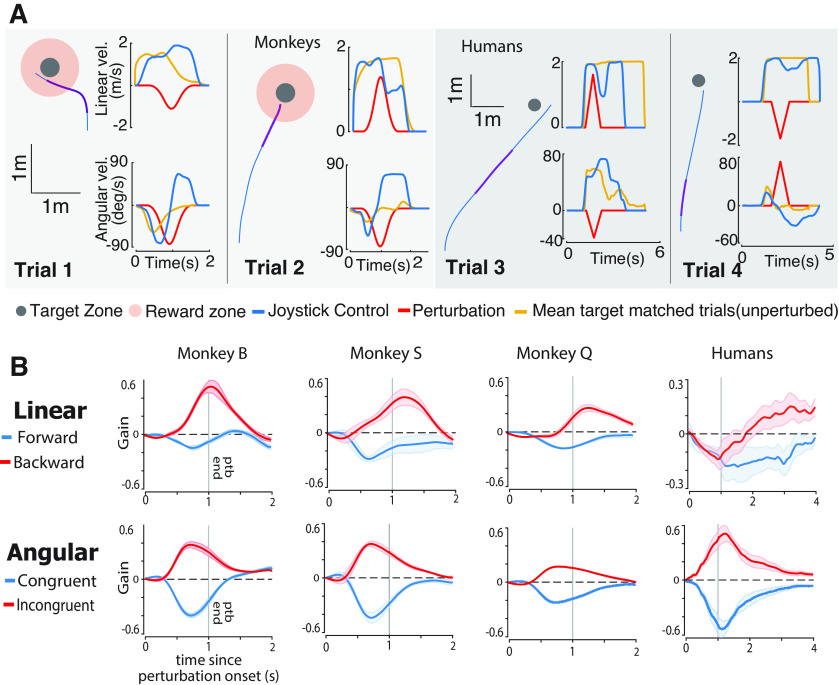
The dynamic profile of responses to perturbations. ***A***, Left, Aerial view of the trajectories for four trials with different perturbation profiles. Purple segments of the trajectories correspond to the perturbation period. Right, Time course for the linear (top) and angular (bottom) velocities. Red line, Perturbation velocity profile; blue line, joystick-controlled velocity of the current trial; yellow line, mean trajectory for 50 target-matched trials without perturbations. ***B***, Average linear (top) and angular (bottom) components of individual monkey (from left, columns 1–3) and human (average across individuals) responses to the perturbations, aligned to perturbation onset. Each component is grouped based on whether the perturbation drives the subject toward (blue, forward/congruent rotation) or away (red, backward/incongruent rotation) from the target. The response is normalized and expressed in units of the perturbation amplitude. Gray vertical line denotes the end of perturbation (see above, Materials and Methods).

In summary, the end points of subjects' trajectories with perturbations are significantly different from those expected from an uncompensated behavior, demonstrating that both macaques and humans can integrate optic flow effectively. To better understand how subjects compensated for perturbations, we investigated the dynamic profile of the subjects' responses as a function of the direction and magnitude of the perturbations. Representative example trials show different steering responses for forward versus backward perturbations. Subjects responded to backward perturbations by increasing their linear speed and extending travel duration (Fig. 4*A*, Trials 1 and 4). In contrast, subjects responded to forward perturbations by decreasing their speed and reducing their travel time (Fig. 4*A*, Trials 2 and 3). For the angular component, subjects rotated in the opposite direction to the angular velocity of the pertubation, even when the perturbation would have brought them closer to the target (Fig. 4*A*).

We grouped subjects' responses based on whether the perturbation pushed subjects toward (forward/congruent) or away (backward/incongruent) from the target and computed average responses separately for the linear and angular perturbations (Fig. 4*B*; see above, Materials and Methods). Dynamic responses to perturbations were estimated for each subject by computing the average deviation of self-motion during the perturbed trials from unperturbed target-matched trials within a time window of 2 s from the perturbation onset, normalized by the perturbation amplitude on that trial. The sign of the response denotes the direction of the response relative to the perturbation direction, and the amplitude indicates the strength of the compensation.

The amplitude of the dynamic compensation was larger for perturbations that pushed monkeys backward (away from the target) compared with those that pushed them forward (toward the target, 0.24 ± 0.11 SD vs 0.44 ± 0.17; *t* test, *p* = 0.002). In contrast, angular compensation was comparable for rotations toward (congruent) and away (incongruent) from the target (0.39 ± 0.14 vs 0.34 ± 0.13, *p* = 0.31; Fig. 4*B*). The reason for symmetric effects in the angular domain is that on most trials, monkeys were nearly done rotating toward the target by the time the perturbation arrived (Fig. 4*A*, example trials) and therefore did not really benefit from congruent perturbations. Qualitatively similar findings were seen for human subjects. For angular responses, humans rotated in the opposite direction of the angular velocity of the pertubation with response amplitudes that were similar for congruent and incongruent rotations (0.57 ± 0.15 vs 0.56 ± 0.15, *p* = 0.84). For linear responses, humans were more conservative, as they slowed down at the time of perturbation onset (Fig. 4*A*) and later producing the adequate response by increasing/decreasing their velocity or travel time depending on the perturbation direction. The response amplitude of human subjects was comparable for forward and backward perturbations (0.19 ± 0.08 vs 0.16 ± 0.09, *p* = 0.64; Fig. 4*A*,*B*). It is likely that the tendency of humans to slow down immediately after the perturbation onset allowed them to more effectively decouple the effects of self-motion and external perturbations on optic flow and compensate better than monkeys.

Nevertheless, despite these small differences between macaques and humans, these results indicate that subjects do use optic flow to dynamically adjust the speed and duration of steering according to the perturbation properties.

### Subjects adjust their velocity according to joystick control gain

**Figure 5.**
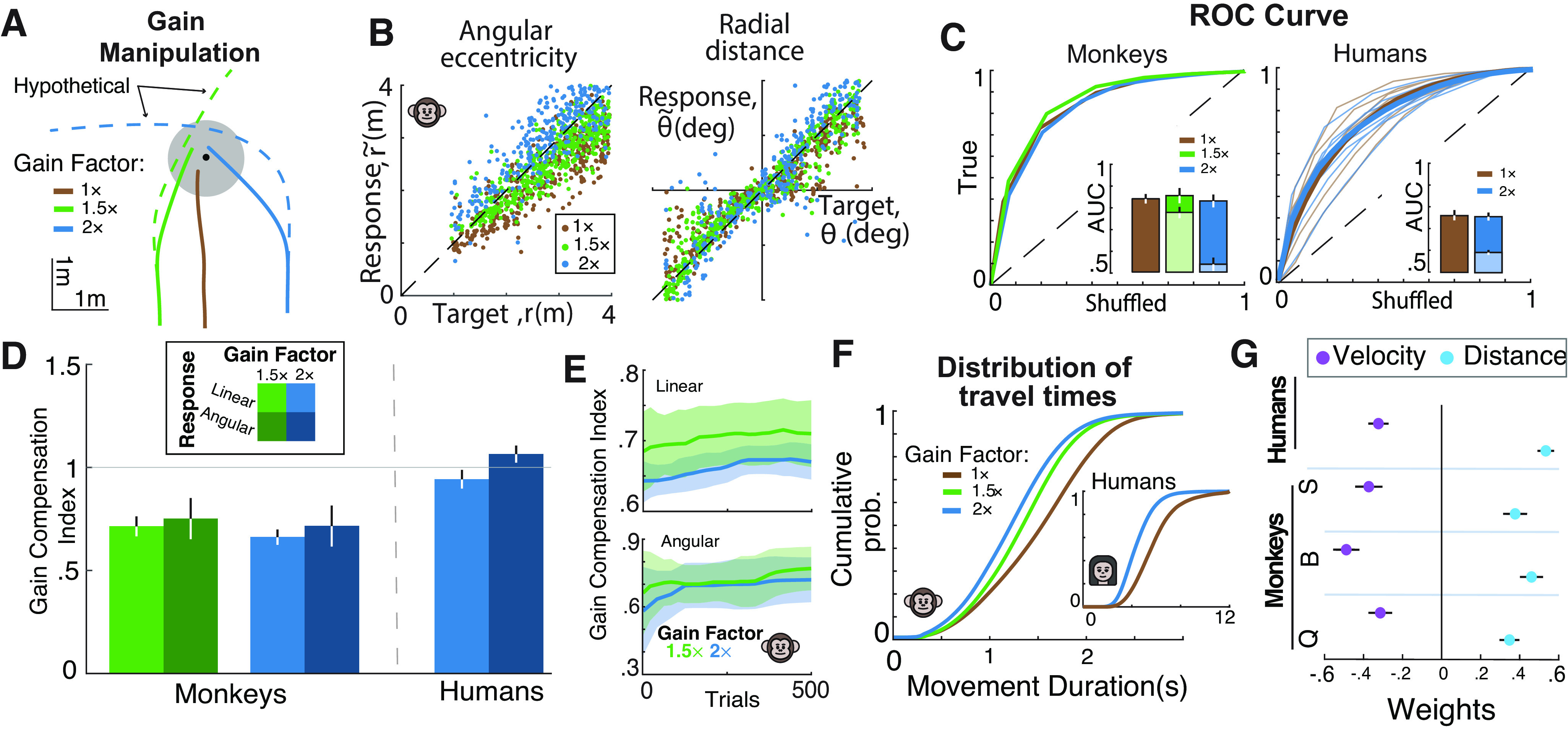
Subjects adjust their responses to different values of joystick controller gain. ***A***, Steering trajectories to an example target (black dot) with joystick gain factor ×1 (solid brown curve), ×1.5 (solid green curve), and ×2 (solid blue curve). Dashed green and blue curves represent the hypothetical (uncompensated) trajectories with no compensation. ***B***, Radial (left) and angular (right) responses of an example monkey during trials from different gain conditions (brown, 1×; green, 1.5×; blue, 2×). ***C***, ROC curves of all monkeys (left) and humans (right) obtained by plotting the proportion of correct trials against the corresponding chance-level proportion from shuffled data. Inset, Area under the curve for real data (dark blue/green) and uncompensated trajectories (light blue/green). ***D***, Bar plot of the mean gain compensation index for monkey and human subjects. Green and blue colors represent gain factor ×1.5 and 2, respectively, and color saturation denotes response type (light color, linear; dark color, angular). Gray horizontal line denotes GCI value of 1. ***E***, Mean gain compensation index in monkeys as a function of trial number for linear (top) and angular (bottom) response and gain factor 1.5 (green) and 2 (blue). Error bars are ±1 SEM. ***F***, Cumulative distribution of travel time for all monkeys (inset, humans) under different gain conditions. ***G***, Coefficients of regression model [$\text{log}(T)=w_{r}\text{log}(r)+w_{\upsilon }\text{log}(\upsilon )$] capturing the effects of gain and distance on travel time. Filled circles represent regression weights of distance (cyan) and gain (purple).

In another version of the task, we manipulated the mapping between actions and their consequences by altering the gain of the joystick controller (Fig. 5*A*). In monkeys, the joystick control gain was altered to vary among 1×, 1.5×, and 2× in separate blocks comprising 500 trials each. In humans, the gain factor varied randomly between 1× and 2× on different trials. To assess how much subjects adjust their responses to the different gain manipulations, we once again compared behavioral responses with hypothetical uncompensated trajectories (Fig. 5*A*, dashed lines), computed by multiplying linear and angular responses during gain 1× trials by the altered gain factor (1.5× or 2×). If subjects ignored the sensory feedback from optic flow cues, their steering would not be significantly different from the uncompensated responses.

To test this, we computed multiplicative response biases by regressing the radial distance $\tilde{r}$ and angular eccentricity $\tilde{\theta }$ of the subjects' final position against the initial target distance r and angle θ (Fig. 5*B*). For monkeys, both linear and angular biases increased with gain factor (mean ± SD); linear bias 0.82 ± 0.03 (gain 1), 0.93 ± 0.08 (gain 1.5), 1.09 ± 0.08 (gain 2; one-way ANOVA, *p* < 10^−6^); angular bias, 0.73 ± 0.05 (gain 1), 0.81 ± 0.12 (gain 1.5), 0.94 ± 0.24 (gain 2; one-way ANOVA, *p* = 0.02). Notably, in all cases, the biases of the animals were significantly smaller than the uncompensated response biases, suggesting that monkeys successfully compensated for changes in joystick gain [linear, paired *t* test, *p* < 10^−6^ (1.5×), *p* < 10^−6^ (2×); angular, *p* < 10^−3^ (1.5×), *p* < 10^−3^ (2×)]. Additionally, the area under the curve (Fig. 5*C*) was comparable for all joystick gains; 0.83 ± 0.03 (gain 1×), 0.86 ± 0.06 (gain 1.5×), and 0.82 ± 0.05 (gain 2×; one-way ANOVA: *p* = 0.12) and significantly different from the uncompensated case (gain 1.5×, *p* = 0.002; gain 2×, *p* < 10^−6^). Combined, these results suggest that monkeys adjust their steering according to the joystick control gain, further supporting the hypothesis that they take visual sensory input into account when choosing their actions.

Human subjects demonstrated even more ideal behavior. There was no difference in either the linear bias [mean ± SD, 1.33 ± 0.29 (gain 1×) vs 1.27 ± 0.25 (gain 2×), *p* = 0.24] or the angular bias [1.6 ± 0.26 (gain 1×) vs 1.68 ± 0.28 (gain 2×), *p* = 0.23], yet bias differed from the uncompensated case responses (linear, *p* < 10^−3^; angular, *p* < 10^−3^). Similarly, there was no gain dependence in the measured AUC; 0.77 ± 0.06 (gain 1×) and 0.76 ± 0.06 (gain 2×, *p* = 0.37), but both were higher than the uncompensated case (*p* < 10^−3^).

To further quantify subjects' responses to the different control gains across humans and monkeys, we computed a GCI, which represents the extent of compensation relative to baseline (gain factor 1×, GCI of 0 means no compensation, GCI of 1 means perfect compensation). Monkey GCIs averaged the following ± SE: linear/angular, 0.71 ± 0.05/0.77 ± 0.09 (gain 1.5×) and 0.67 ± 0.03/0.72 ± 0.09 (gain 2×). Human GCIs were closer to ideal, 0.94 ± 0.04/1.06 ± 0.03 (gain 2×; Fig. 5*D*).

Next, we investigated how the compensation to the different gain factors evolved over the time course of the 500-trial blocks. Remarkably, within the first 20 trials the GCI was significantly different from zero for both linear and angular responses [mean GCI ± SE; linear, 0.67 ± 0.03 (gain 1.5×), *t* test, *p* < 10^−3^, 0.64 ± 0.02, *t* test, *p* < 10^−3^ (gain 2×); angular, 0. 69 ± 0.05, *t* test, *p* < 10^−3^ (gain 1.5×), 0.6 ± 0.06, *t* test, *p* < 10^−3^ (gain 2×); Fig. 5*E*], showing that monkeys adjusted their responses promptly to the new gain factors. Furthermore, the efficacy of compensation slightly increased throughout the block as shown by the correlation (see above, Materials and Methods) of the GCI with the trial number [Pearson's *r* ± SE; linear, 0.43 ± 0.04, *p* = 0.005 (gain 1.5×), 0.6 ± 0.03, *p* < 10^−3^ (gain 2×); angular, 0.43 ± 0.03, *p* = 0.005 (gain 1.5×), 0.3 ± 0.03, *p* = 0.03 (gain 2×)], although the magnitude of increase was quite small.

Evidence for compensation was also seen in travel time. For humans, the mean travel time was lowest for highest gain [4.48 ± 0.84 s (gain 2×) vs 5.89 ± 1.39 s (gain 1×; paired *t* test, *p* < 10^−3^)]. This was also true for monkeys [1.35 ± 0.17 s (gain 1.5×) and 1.31 ± 0.19 (gain 2×) vs 1.66 ± 0.23 s (gain 1×; one-way ANOVA, *p* < 10^−3^)]. Thus, both humans and monkeys adapted to the different gain values by adjusting their travel duration appropriately (Fig. 5*F*).

Collectively, these results show that monkeys and humans adapt their responses to the changing joystick gain, supporting the hypothesis that subjects perform the task successfully by integrating optic flow. Even so, the sparse optic flow may not be the only information about the subjects' spatial location in the virtual environment relative to the position of the target. Subjects could also partially incorporate predictions from an efference copy of their joystick movements.

To test the hypothesis that the subjects' navigation strategy is based on optic flow integration, we contrasted it with pure time integration. To quantify the relative dependence on the two strategies, we took advantage of the lawful relationship among travel time, distance, and velocity [velocity = distance/time]. Accordingly, we simultaneously regressed the travel time against initial target distance (*r*) and mean velocity (υ) in the log space across trials [$\text{log}(T)=w_{r}\text{log}(r)+w_{\upsilon }\text{log}(\upsilon )$; see above, Materials and Methods]. The travel time of an ideal path integrator would depend on changes in both distance and gain, with $w_{r}=1\mathrm{and}$ $w_{\upsilon }$ = −1. In contrast, the alternative strategy of pure time integration would predict weights $w_{r}=1$ and $w_{\upsilon }=0$ (no dependence on velocity). Across all subjects, the regression weight on velocity, $w_{\upsilon }$, was significantly different from zero in all monkey and human subjects [95% confidence interval (CI) of regression weight]; Monkey Q [−0.34, −0.28], Monkey B [−0.52, −0.46], Monkey S [−0.41, −0.34], Humans [−0.35, −0.30]; Fig. 5*G*. The weight on target distance w_r,_ was positive and different from zero; Monkey Q [0.32, 0.37], Monkey B [0.43, 0.49], Monkey S [0.34, 0.41], Humans [0.51, 0.55]; Fig. 5*G*. Notably, when this analysis was restricted to rewarded trials only, $w_{\upsilon }$, was closer to −1, Monkey Q [−0.99, −0.91], Monkey B [−1.01, −0.92], Monkey S [−1.02, −0.95], Humans [−0.94, −0.88], and $w_{r}$ was closer to 1, Monkey Q [0.84, 0.92], Monkey B [0.83, 0.91], Monkey S [0.84, 0.92], Humans [0.94, −0.99]. This analysis of the responses to gain manipulation clearly supports the hypothesis that subjects perform the task by integrating optic flow.

### Optic flow density affects task performance

**Figure 6.**
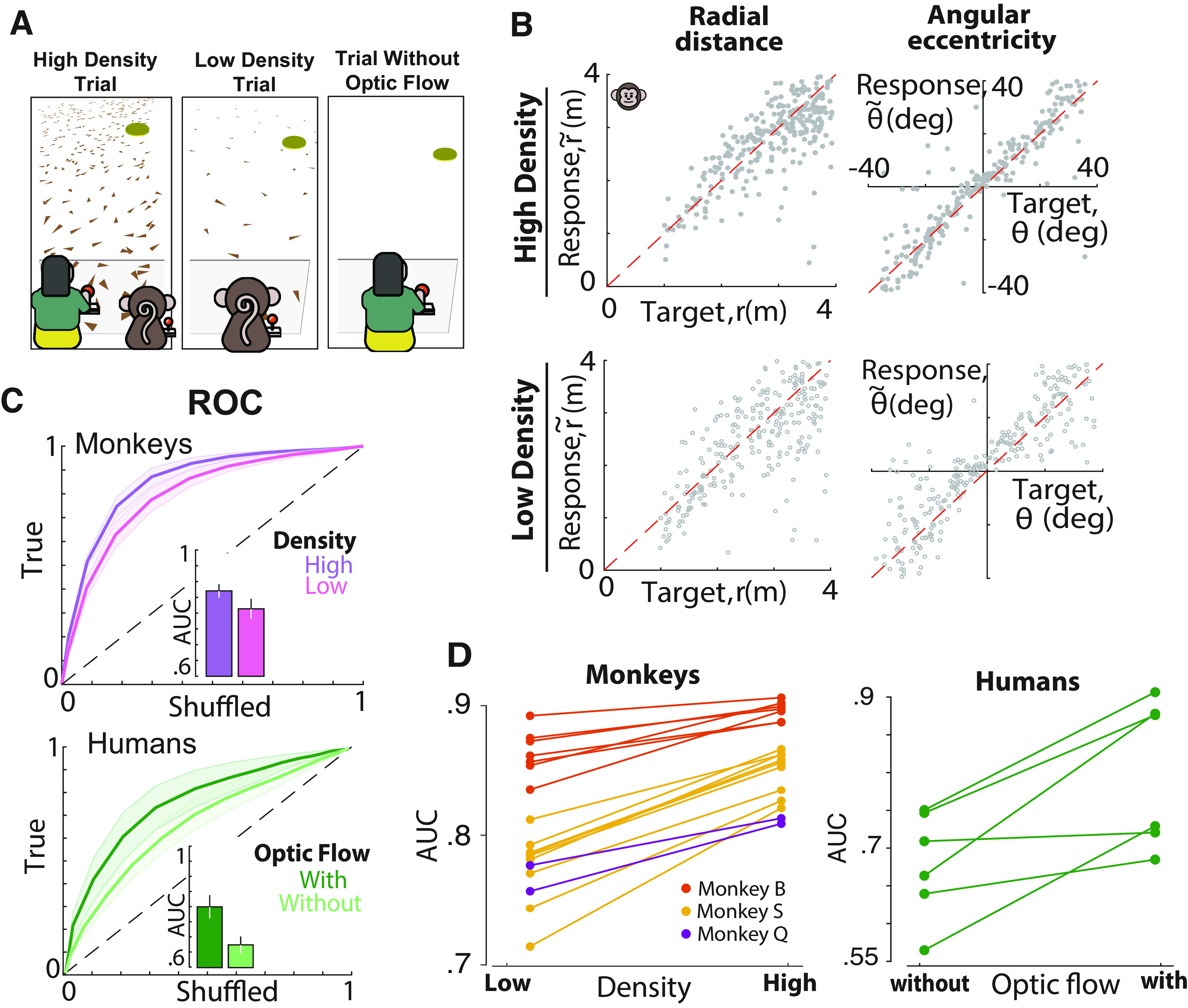
Optic flow density affects subjects' performance. ***A***, Illustration of the types of optic flow density conditions used. Left, Trial conditions where ground elements occur at high density, a condition that was consistent for both monkeys and humans. Middle and right, Trials with sparse (monkeys only) and trials without (humans only) ground plane elements, respectively. ***B***, Radial and angular response of an example monkey with high- (top) and low-density (bottom) optic flow cues. ***C***, Top, ROC curves for all the monkeys for low- (pink) and high-density (purple) optic flow cues. Bottom, ROC curves for all the human subjects for trials with (dark green) and without (optic flow) optic flow cues. Shaded area represents SEM for all the human and monkey subjects, respectively. Inset, AUC. ***D***, Pairwise comparison of the AUC for all the monkeys (left) and human subjects (right).

A final manipulation involved changing the informativeness of optic flow by varying the density of the ground plane elements between two possible values (sparse and dense for monkeys, with and without optic flow for humans; Fig. 6*A*). If subjects rely on optic flow integration to navigate, different values of ground plane density would have an impact on the subjects' responses. Indeed, the overall response variability was much larger for low-density conditions across monkey subjects for both linear (SD ± SE, high density, 0.56 ± 0.05 m; low density, 0.68 ± 0.05 m; *t* test, *p* < 10^−3^) and angular (high density, 9 ± 0.8°, low density, 10 ± 0.8°; *p* < 10^−3^) responses (Fig. 6*B*). Likewise, in human subjects, the removal of optic flow increased the SD of linear (with optic, 1.11 ± 0.08 m; without optic flow, 1.37 ± 0.07 m; *t* test, *p* = 0.03) and angular (with optic flow, 14.15 ± 3.1°, without optic flow, 40.83 ± 3.3°; *p* = 0.01) responses. Altering the density of optic flow affected subjects' accuracy by increasing the absolute error in monkeys (mean Euclidian error ± SD, high density, 0.65 ± 0.2 m; low density, 0.8 ± 0.22 m; *t* test, *p* < 10^−6^) and humans (with optic flow, 1.68 ± 0.91 m; without optic flow, 2.5 ± 0.73 m; *t* test, *p* = 0.003; Note: All trials, not just those rewarded, were included). This difference was also reflected in ROC analysis (Fig. 6*C*,*D*; AUC ± SD, high density, 0.85 ± 0.06; low density, 0.79 ± 0.08, *p* < 10^−6^). Similarly, removal of optic flow cues in humans decreased AUC from 0.8 ± 0.09 (optic flow) to 0.67 ± 0.07 (no optic flow; *t* test, *p* = 0.012). Once again, these results collectively suggest that the subjects rely heavily on optic flow to navigate to the target.

**Figure 7.**
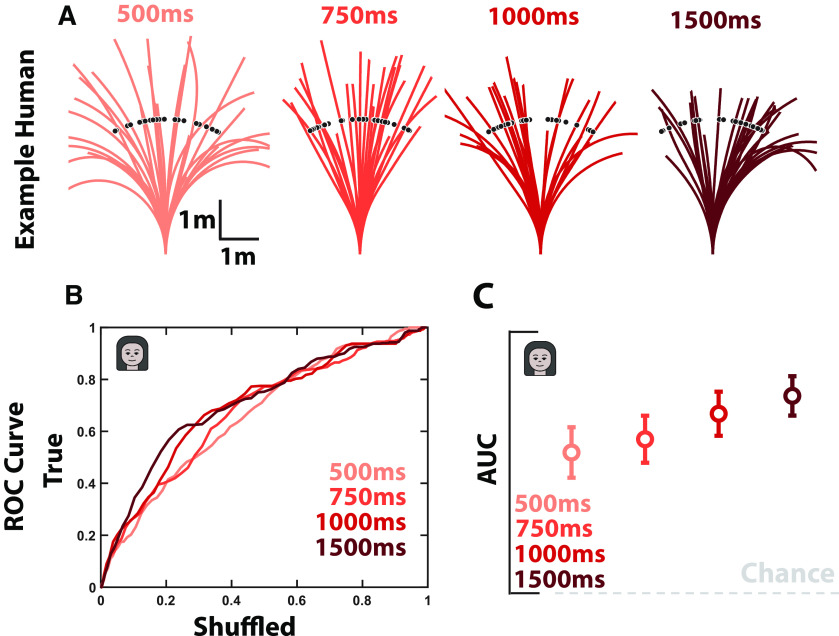
Humans integrate optic flow throughout the duration of their trajectories. ***A***, Target locations (black dots) and trajectories for a subset of trials in an example subject. As the presentation of optic flow information increased in duration (red gradient, left to right, 500, 750, 1000, and 1500 ms) the subject's overshooting of targets became less drastic. This is in line with a known prior for slow speeds in humans during this task (Lakshminarasimhan et al., 2018a; Noel et al., 2020), given that at lower optic flow presentation durations the trajectory ought to be further driven by the prior. ***B***, ROC curves for all subjects (thin line) and their average (thick line) as a function of optic flow duration. ***C***, Mean AUC of the ROC. Error bars are ±1 SEM.

In a final variant of this task (Fig. 7), we also manipulated the duration of the ground plane optic flow. Specifically, in humans (separate cohort, *n* = 11) optic flow was presented for 500, 750, 1000, or 1500 ms from trial onset (randomly intermixed across trials). We reasoned that if humans were not integrating optic flow across the duration of their trajectories (median duration ∼2000 ms) but instead were using the optic flow to initially adjust their internal models (e.g., their velocity estimate) and then navigated by dead reckoning, then their performance would not continuously improve with increasing optic flow durations. Performance, as measured by the area under the ROC, continuously improved with increasing optic flow durations (one-way ANOVA, *F*_(3,36)_ = 2.74, *p* < 0.05, Bonferroni-corrected *post hoc t* test, all *p* values < 0.05; Fig. 7), supporting the hypothesis that observers do indeed integrate optic flow over the entire duration of their trajectories.

In summary, we found that both macaques and humans were able to compensate for dynamic perturbations of internal state by using optic flow cues. In addition, subjects used optic flow to adjust their control and adapt to uncued changes in joystick gain. In both cases, the observed compensation was less than ideal, suggesting that subjects may also rely on an internal model of the control dynamics to some extent. Consistently with this, we found that removing optic flow cues decreased accuracy but did not completely blunt performance. Most critically, humans integrate optic flow throughout the duration of their trajectories.

## Discussion

Using three independent manipulations of the same basic navigation task, we showed that both monkey and human subjects rely on optic flow to navigate to the flashed target. Specifically, we introduced external perturbations to the subjects' movement, varied the control gain of the joystick, and altered the density (and timing) of the optic flow to test whether the subjects integrate optic flow information to infer their self-location relative to the target. We found that subjects adjusted their steering velocity to compensate for external perturbations, adapted to joystick control gain changes, and showed degraded performance as the sensory uncertainty of the optic flow was decreased (monkeys) or eliminated (humans).

Human navigation research has long taken for granted that path integration by integration of optic flow is feasible, but it never explicitly tested this hypothesis using naturalistic paradigms. Most previous studies used experimental paradigms that have artificial constraints, such as passive translation (Klatzky et al., 1998; Jürgens and Becker, 2006; Petzschner and Glasauer, 2011; Campos et al., 2012; Tramper and Medendorp, 2015), discretized decision and actions (Ter Horst et al., 2015; Chrastil et al., 2016; Koppen et al., 2019), or restricted movements to a one-dimensional track (Frenz and Lappe, 2005; Frenz et al., 2007; Campbell et al., 2018). In contrast, real-world navigation is an active process with rich temporal dynamics that typically takes place in two dimensions. By incorporating all three features into the task, our findings validate the utility of optic flow for navigation in the absence of landmarks.

The approach used here also has implications for studying sensory evidence accumulation in general. Traditionally, evidence accumulation has been studied using paradigms in which subjects passively view a noisy stimulus with predefined dynamics, integrate evidence for a period of time, and then report their decision at the end of the trial (Gold and Shadlen, 2007; Kiani et al., 2013). In such tasks, the latent dynamics are not under the subject's control. The active nature of the task used here highlights fundamental aspects of behavior such as the closed-loop interaction between sensation and action, the travel cost, and the uncertainty about the dynamics of the world, which are intractable in passive tasks. In past studies, passive tasks had been used to test the contribution of optic flow in navigation (Frenz and Lappe, 2005; Lappe et al., 2007); however, large inaccuracies (biases) persisted in performance despite feedback (Petzschner and Glasauer, 2011; McManus et al., 2017). On the other hand, more recent studies support an increased granularity of information sampling (Voigts et al., 2015) during active tasks, as well as an improvement in subjects' ability to recognize irregularities during evidence accumulation (Górska et al., 2018). These findings are congruent with our results, showing that subjects compensate almost perfectly for unpredicted perturbations of optic flow and raise the question of whether the estimation of unpredicted displacements would be comparable under passive movement. Although a direct comparison to previous passive paradigms is not straightforward because of task differences, we could speculate that active behavior plays a fundamental role in the accurate learning of an internal model initially, which significantly affects task performance. However, this is a question to be addressed in future studies with an adequate task design that will allow effective comparison.

The task used in this study offers a way to study evidence accumulation in a more naturalistic setting, where observations and internal model predictions are continuously combined to estimate self-location. In this study, we focused on the effect of observations on evidence accumulation; it would also be informative to explore when evidence about the internal model predictions are manipulated. Specifically, increasing the process noise of motor control would increase the extent to which subjects rely on sensory input for evidence accumulation and vice versa. Recent rodent decision-making research has started moving in this direction by training animals to continuously accumulate evidence while navigating in a maze (Pinto et al., 2018; Nieh et al., 2021); however, the range of possible actions in such tasks is still limited. In contrast, subjects here could use both linear and angular components of optic flow to navigate toward a continuum of possible target locations. The moderate complexity of this task can more accurately capture real-world dynamics and allow for future exploration of sensory evidence accumulation and decision-making with fewer restrictions than traditional binary tasks, without sacrificing the ability to manipulate task variables (Noel et al., 2021).

Our findings support an optic-flow-based navigation strategy that is conserved across humans and monkeys, thus paving the way for the study of neural mechanisms of path integration in monkeys. There is already a well-documented hierarchy of regions in the macaque brain that are involved in processing optic flow (Britten, 2008), including a representation of both linear and angular velocity in the posterior parietal cortex (Avila et al., 2019). Analyzing the relationship of neural responses in those areas to the estimates of the animal during binary decision tasks have helped better the understanding of the feedforward mechanisms underlying heading perception (Pitkow et al., 2015; Lakshminarasimhan et al., 2018b). Analyzing neural recordings during richer tasks such as the one used here calls for more sophisticated tools (Balzani et al., 2020; Kwon et al., 2020; Wu et al., 2020) but will likely shed light on the recurrent mechanisms that underlie more dynamic computations of perceptual decision-making in closed-loop dynamic tasks.

It must be noted that results from all three manipulations were consistent with a strategy that combines sensory feedback control based on optic flow cues, and predictive control based on an internal model of the dynamics. Such a combination has been extensively reported in the context of motor control tasks (Wolpert et al., 1995; Wolpert and Ghahramani, 2000). Thus, similar principles may underlie control of limb movements and visuomotor control using external affordances, such as driving a car. Given the rich experimental evidence for the role of cerebellum in constructing internal models (Ito, 2008), cerebellar targets to the posterior parietal cortex (Bostan et al., 2013) may prove important in the fine line between internal model predictions and sensory feedback signals such as optic flow.
